# The Leaf Essential Oil of *Myrtus communis* subsp. *tarentina* (L.) Nyman: From Phytochemical Characterization to Cytotoxic and Antimigratory Activity in Human Prostate Cancer Cells

**DOI:** 10.3390/plants12061293

**Published:** 2023-03-13

**Authors:** Claudia Giuliani, Roberta Manuela Moretti, Martina Bottoni, Laura Santagostini, Gelsomina Fico, Marina Montagnani Marelli

**Affiliations:** 1 Department of Pharmaceutical Sciences, Università degli Studi di Milano, Via Mangiagalli 32, 20132 Milan, Italy; 2 Ghirardi Botanical Garden, Department of Pharmaceutical Sciences, Università degli Studi di Milano, Via Religione 25, 25088 Toscolano Maderno, Italy; 3 Department of Pharmacological and Biomolecular Sciences, Università degli Studi di Milano, Via Balzaretti 9, 20133 Milan, Italy; 4 Department of Chemistry, Università degli Studi di Milano, Via Golgi 19, 20133 Milan, Italy

**Keywords:** *Myrtus communis* subsp. *tarentina* (L.) Nyman, essential oil, secretory cells, botanical garden, prostate cancer cell lines, antitumor potential, apoptosis, cell migration

## Abstract

The aim of this study was to investigate the chemical profile and the cytotoxic activity in two castration-resistant prostate cancer (CRPC) cell lines of the leaf essential oil in *Myrtus communis* subsp. *tarentina* (L.) Nyman (EO MT), which was cultivated at the Ghirardi Botanical Garden (Toscolano Maderno, Brescia, Italy). The leaves were air-dried and extracted by hydrodistillation with a Clevenger-type apparatus, and the EO profile was characterized by GC/MS. For the cytotoxic activity investigation, we analyzed the cell viability by MTT assay, and the apoptosis induction by Annexin V/propidium iodide assay/Western blot analysis of cleaved caspase 3 and cleaved PARP proteins. Moreover, the cellular migration was analyzed by Boyden’s chamber assay and the distribution of actin cytoskeleton filaments by immunofluorescence. We identified 29 total compounds; the main compound classes were oxygenated monoterpenes, monoterpene hydrocarbons, and sesquiterpenes. The main constituents were α-pinene, α-humulene, α-terpineol, durohydroquinon, linalool, geranyl acetate, and β-caryophyllene. We found that EO MT was able to reduce cellular viability, activating an apoptotic process, and to decrease the migratory capacity of CRPC cells. These results suggest that it might be interesting to further investigate the effects of single compounds present in EO MT for their possible use in prostate cancer treatment.

## 1. Introduction

Previous studies in the literature have testified to the great interest that the essential oils (EOs) or extracts of *Myrtus communis* L. (Myrtaceae), with reference to the nominal subspecies, generate for their potential anticancer activity [1,2,3,4,5,6,7].

Myrtle EO profiles have been extensively studied [8,9,10,11,12,13,14], and in Italy, previous contributions refer to samples growing in the regions of Sardinia, Sicily, Campania, and Liguria [11,12,13,14,15,16,17,18]. This prompted us to firstly analyze the EO profile of the nominal subspecies [19] cultivated in Northern Italy at the Ghirardi Botanical Garden (Toscolano Maderno, Brescia, Lombardy) of the University of Milan. In this study, we examined *Myrtus communis* subsp. *tarentina* (L.) Nyman, preserved at the same study site and on which previous phytochemical studies are lacking.

Two subspecies are formally recognized within *M. communis* [20]. The diagnostic characters refer to the total plant size and the leaf, branch, and fruit features. *M. communis* subsp. *tarentina* differs from the nominal species in its limited height, being up to two meters high, the closely spaced and smaller leaves no longer than 2 cm, the sturdy and small branches, and the globular berries [20]. Its distribution range includes Corse, France, Italy (Sardinia), Kriti, Portugal, Spain, and the countries of the Balkan Peninsula [20]. 

Wild populations display a large degree of polymorphism [21,22]. This led previous authors to study genetic diversity that was addressed in different ways by examining morphological traits, anatomy, biochemical markers, and DNA-based markers [21,22].

Concerning the data in the literature on the micromorphology, previous investigations focus on the nominal subspecies and describe the structure, ontogeny, and histochemistry of the secretory cavities of leaves, shoots, and flowers by means of both light and electronic microscopy [19,23,24,25,26].

The discovery of natural products of plant origin and their introduction on the market in combination with synthetic drugs has made it possible to cure many diseases [27]. Regarding natural molecules with therapeutic potential, in recent years, interest in EOs as preventive or potential therapeutic agents against a wide variety of pathologies, including cancer, has increased [28].

Prostate cancer represents the second most common solid tumor in men and the fifth cause of cancer-related death [29]. The growth of prostate cancer in its early stages is supported by androgens, and androgen deprivation therapy (ADT) represents the gold standard therapy of the disease [30]. However, a high percentage of patients develop castration-resistant prostate cancer (CRPC). At this stage, the cells are unresponsive to ADT and effective treatment options are limited. Therefore, the discovery of new effective molecules for the development of new therapeutic strategies suitable for counteracting the growth and spread of the tumor in the most aggressive phases is mandatory [31]. 

We performed a multidisciplinary research approach on the EO of *M. communis* subsp. *tarentina* (EO MT) to: (a) analyze the composition of the EO MT obtained from dried leaves collected in July 2018 and (b) evaluate the anticancer potential of EO MT in two CRPC cell lines (DU145 and PC3 cells). We also describe for the first time the micromorphology and histochemistry of the secretory cavities of leaves, to clarify whether there was consistency regarding the literature information about the nominal subspecies.

The present work is part of a wider project entitled *Botanic Garden, factory of molecules…work in progress* (Call of the Lombardy Region Lr 25/2016, year 2021), aimed at investigating a selection of species preserved at the Ghirardi Botanical Garden through a multi-level study approach: micromorphological, phytochemical, and concerning the ecology and biological activity of the secondary metabolites. This project is, in turn, in continuity with the project proposal entitled *BOHEMY: From BOtanical garden to human HEalth: MYrtle as source of novel bioactive compounds* presented within the SEED 2019 Call of the University of Milan.

## 2. Results and Discussion

### 2.1. Phytochemical Characterization

The composition of the leaf EO from *M. communis* subsp. *tarentina* was determined to investigate any possible correlation between EO components and their activity on prostate cancer cells.

The EO, obtained with a yield equal to 0.47%, resulted as mainly composed of oxygenated monoterpenes (44.91%), followed by monoterpene hydrocarbons (20.49%) and sesquiterpenes (17.84%), while the other classes showed a relative percentage lower than 9.0% (Table 1). 

The main compounds were: α-pinene (**1**, 17.73%), α-humulene (**22**, 12.39%), α-terpineol (**10**, 7.36%), durohydroquinon (**25**, 7.26%), linalool (**6**, 6.16%), geranyl acetate (**18**, 5.92%), β-caryophyllene (**20**, 5.17%), methyl eugenol (**19**, 4.34%), humulene epoxide II (**28**, 3.19%), and an undetermined compound (**3**, 11.96%). 

Ten compounds (**4**, **5**, **9**, **11**, **12**, **14**, **16**, **17**, **26**, **29**) exhibited relative abundances in the range 1.0–2.0%, while the remaining nine identified compounds were found with percentage values between 0.1 and 1.0%.

The comparison between *M. communis* subsp. *communis* (MC) and *M. communis* subsp. *tarentina* (MT), gathered at the same collection time (July 2018) at the same study site (Ghirardi Botanical Garden), and therefore grown under the same parameters of cultivation and under the same pedo-climatic conditions, highlighted two different quali-quantitative compositions. Moreover, the plant material was subjected to similar conservation procedures, i.e., air-drying at room temperature. EO MT appeared richer in compounds than MC (**29** versus **25**, respectively). The two profiles shared the presence of the following compounds: α-pinene (**1**) and β-pinene (**2**), γ-terpinene (**4**), α-terpinolene (**5**), linalool (**6**), 4-terpineol (**9**), α-terpineol (**10**), linalyl acetate (**11**), β-caryophyllene (**20**), α-humulene (**22**), and durohydroquinon (**25**), the relative abundances of which differed greatly. Specifically, α-pinene (**1**) and linalool (**6**) resulted as highly abundant in MC compared to MT (41.6% vs. 17.73% and 18.2% vs. 6.16%, respectively). EO MT was instead characterized by a higher content of α-terpineol (**10**, in a three-times greater amount), β-caryophyllene (**20**, in an eight-times greater amount), α-humulene (**22**, in a twenty-times greater amount), and durohydroquinon (**25**, in a ten-times greater amount) compared to MC. 

In the light of these considerations, a literature survey was carried out that aimed to investigate whether an already-known anticancer activity could be ascribed to the detected compounds. A summary of the literature data on all the EO MT compounds, except for those occurring in traces, is presented hereafter.

Regarding α-pinene (**1**) and β-pinene (**2**) (the former more abundant in MC) in MT 17.73% and 0.38%, respectively, synergistic anticancer effects with paclitaxel were documented in small cell lung carcinoma (SCLC) [32]. In addition, Kusuhara and colleagues [33] observed a decrease in melanoma growth in rats placed in fragrant environment with α-pinene. Moreover, in prostate cancer cells this compound reduced cell growth and activated apoptosis [33].

For α-terpineol (**10**) (three-times greater than MC), antioxidant, antitumor, anticonvulsant, and antiulcer activity, with gastroprotective action, were known [34]. Specifically, its anticancer properties consist in the ability to arrest the cell cycle and to induce apoptosis in colorectal cancer, in synergy with camphor and linalyl acetate (**11**). In colon cancer, cytochrome C release promotes apoptosis; in SCLC, α-terpineol (**10**) in association with linalyl acetate (**11**) shows cytotoxic activity by suppressing NF-kB signaling [34].

Geranyl acetate (**18**) (in MT 5.92%—not present in MC) in association with geraniol induced a strong antitumor activity on colon cancer cells, Colo205, activating apoptosis, DNA damage, and cell cycle arrest [35]. It showed anti-inflammatory and oxidative stress reduction activity.

Methyl eugenol (**19**) (4.34% in MT, not in MC) showed anticancer activity in retinoblastoma RB355 through the induction of autophagy [36]. α-Humulene (**20**) (20-times greater than MC) displayed high anti-inflammatory activity in association with caryophyllene [37]. In addition, it showed anticancer activity in hepatocellular carcinoma by inducing mitochondrial apoptosis and promoting caspase 3 activation and PARP cleavage [38]. It also reduced the growth of MCF-7 cancer cells alone and in combination with paclitaxel [39].

Literature data about the anticancer potential of the remaining EO MT compounds are lacking.

### 2.2. Micromorphological Investigation

**Figure 1:**
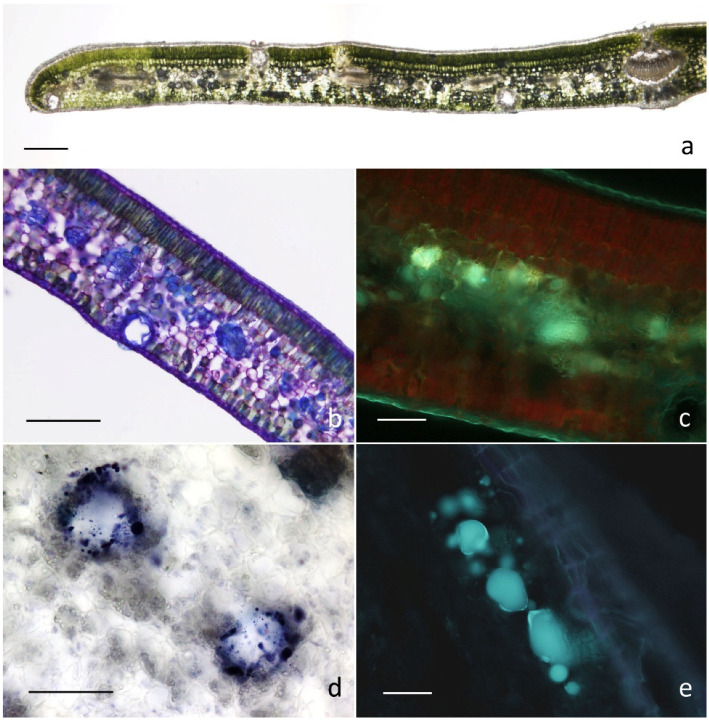
(**a**,**b**) Light microscopy images showing cross section of *Myrtus communis* subsp. *tarentina* leaf, colorless (**a**) and stained with Toluidine blue dye, (**b**). (**c**–**e**) Details of leaf secretory cavities, Fluoral Yellow-088 (**c**), Nadi reagent (**d**), AlCl_3_ (**e**). *Scale bars = 100 μm (**a**–**c**); 50 μm (**d**,**e**)*.

With regards to micromorphology, consistent features emerged among all the examined leaf replicates. The observations evidenced the occurrence of secretory cavities diversely distributed between the palisade and the spongy parenchyma cells (Figure 1). A preferential occurrence was observed at the transition region between the two types of mesophylls or adjacent to the leaf abaxial or adaxial surfaces (Figure 1a,b).

The secretory cavities were globose-spheroidal and displayed a variable diameter ranging up to 40–60 μm. Secretory cells tightly close together and arranged in one-layer covered the lumen of the cavities and released abundant secretory products in the form of small coalescing drops or a large mass (Figure 1c–e). 

Regarding histochemistry, all the dyes specific for lipophilic substances produced positive responses, with special reference to the NADI reagent indicating the copious production of terpenes (Figure 1c,d). The synthesis and release of polyphenols, specifically flavonoids, was also evidenced (Figure 1e). The tests specific for mucopolysaccharides provided invariably negative results. 

These overall observations showed a high level of affinity with the features described for the secretory structures in the nominal subspecies by Giuliani et al. [18] and are in accordance with those reported in the literature for other members of the Myrtaceae family [22,25].

### 2.3. Cytotoxic Activity

#### 2.3.1. Cytotoxic and Proapoptotic Activity of EO MT in CRPC Cells

**Figure 2:**
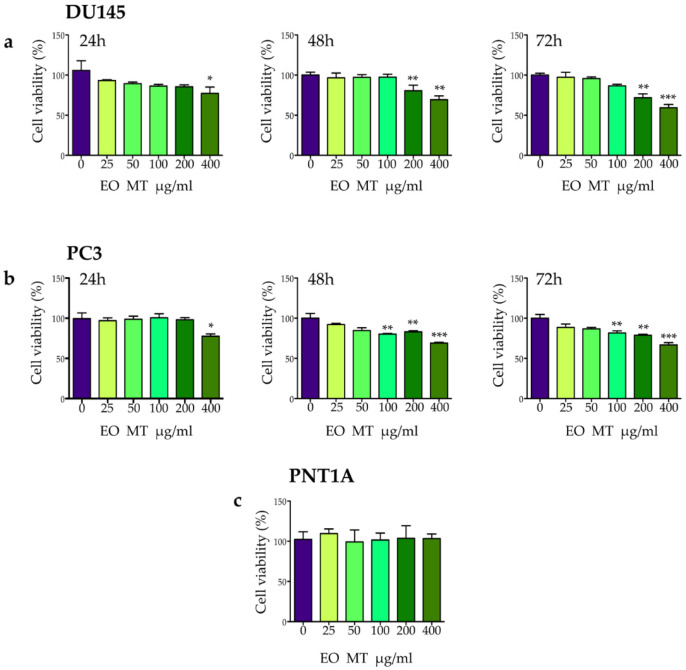
(**a**–**c**). Effect of the leaf essential oil of *Myrtus communis* subsp. *tarentina* (L.) Nyman (EO MT) on DU145 (**a**), PC3 (**b**), and PNT1A (**c**) cell viability. DU145 and PC3 cells were exposed to fresh medium containing the vehicle DMSO (0) or EO MT (25–400 µg/mL). Cell viability was measured by means of MTT assay after 24 h, 48 h, and 72 h. Data represent a mean value (% of control) ± SEM of three independent experiments. Value significant in comparison to controls at least with * *p* < 0.05, ** *p* < 0.001, *** *p* < 0.0001 (one-way ANOVA with Dunnett’s post hoc test). PNT1A cells were exposed to fresh medium or EO MT (25–400 µg/mL) for 48 h and cell viability was measured by MTT assay.

In order to evaluate the potential cytotoxic effects of EO MT on DU145 and PC3 cells, an MTT cell viability assay was conducted. The results obtained after 24 h, 48 h, and 72 h of incubation with increasing concentrations of EO MT (25, 50, 100, 200, 400 μg/mL) are presented in Figure 2a,b. After 48 h and 72 h of EO MT treatment, the dose of 100 µg/mL demonstrated significant decrease in cell viability in PC3 cells, as well as the dose of 200 µg/mL in both cell lines. Moreover, the highest applied concentration of 400 µg/mL was significant starting from 24 h in both cell lines. To assess whether the effect on cell viability of the oil was selective for malignant cells, normal prostate epithelial cells, PNT1A, were treated with increasing concentrations of EO MT (25, 50, 100, 200, 400 μg/mL) for 48 h in a similar manner to the cancer cells. As shown in Figure 2c, the results showed that PNT1A cells were not susceptible to the action of EO MT, also at a higher dose of 400 μg/mL, proving that EO MT is not cytotoxic in normal prostate epithelial cells. Few studies have reported the potential cytotoxicity of *Myrtus communis* L. EO on cancer cell lines. Recently, the cytotoxic effect of *M. communis* L. EO (in a concentration range of 62.5–1000 μg/mL) was investigated in A375 melanoma cells (IC50 580 μg/mL) [40]. 

Moreover, *M. communis* EO showed significant cytotoxicity on neuroblastoma cell line SH-SY5Y (IC50 209.1 μg/mL) [41].

Harassi and colleagues reported a moderate cytotoxicity of two Moroccan *Myrtus communis* L. EOs against P815 cell (IC50 260 μg/mL) [7]. The dose range of EO MT used in our experiments is in agreement with the studies reported above. Moreover, Dolghi and collaborators observed a decrease in cell viability after treatment with *Mentha piperita* L. EO and *Rosmarinus officinalis* L. EO, starting with the concentration of 150 μg/mL to 500 μg/mL in HCT 116 colorectal carcinoma cells [42]. Other authors recently demonstrated a cytotoxic activity of *Tanacetum balsamita* EO on two breast cancer cell lines, MDA-MB-231 and MDA-MB-468, particularly at 100 μg/mL and 200 μg/mL [43]. 

**Figure 3:**
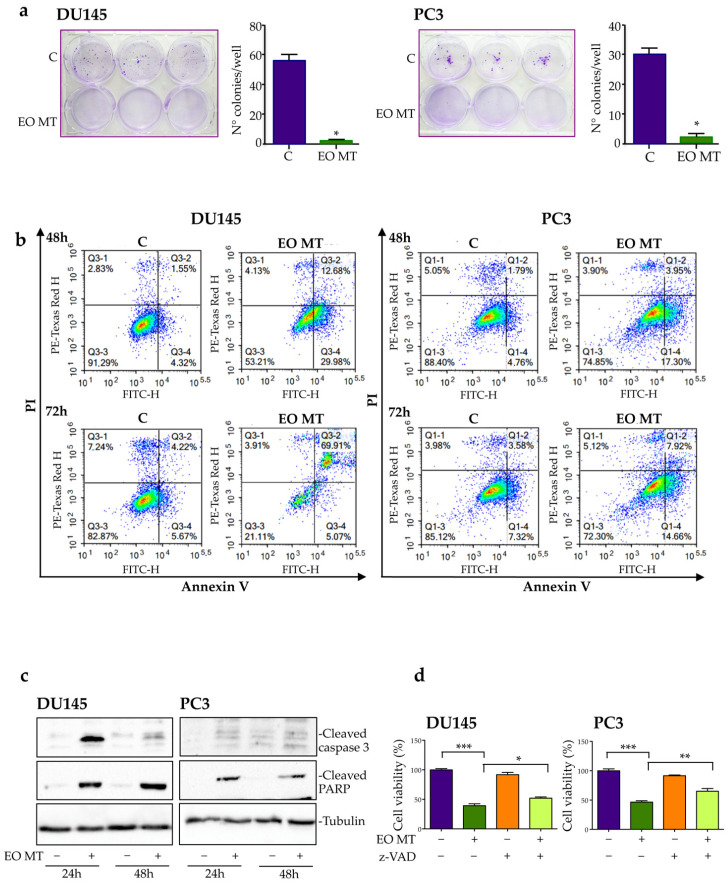
Effect of *Myrtus communis* subsp. *tarentina* (L.) Nyman (EO MT) on clonogenic capacity of DU145 and PC3 cells and apoptosis involvement in its cytotoxic activity. For clonogenic assay (**a**), both cell lines were treated with EO MT (400 µg/mL) for 48 h, and the colony-forming ability was assessed after 12 days of culture. Cell colonies were fixed with methanol stained with crystal violet. Data were expressed as the number of colonies of treated DU145 and PC3 cells in comparison with the no treatment control group (C). Data represent mean values ± SEM and were analyzed by a Student’s *t* test. * *p* < 0.05 vs. C. For flow cytometry analysis (**b**), DU145 and PC3 cells treated with EO MT (400 µg/mL) for 48 h or 72 h were stained with Annexin V/PI and analyzed by Novocyte 3000. In each square, the top-left box represents necrotic cells, the top-right box represents cells in late apoptosis, the bottom-left box represents viable cells, and the bottom-right box represents cells in early apoptosis. A Western blot analysis of apoptosis-related proteins was carried out on control (C) and EO MT (400 µg/mL)-treated cells for 24 h and 48 h (**c**). Tubulin antibody was used as a loading control. One representative of three different experiments performed is shown. An MTT assay was performed to analyze the involvement of apoptosis in the cytotoxic activity of EO MT (**d**), and DU145 and PC3 cells were pretreated with the pan-caspase inhibitor, Z-VAD-FMK (Z-VAD, 50 μM, 4 h), before the EO MT treatment (400 µg/mL, 48 h). Each experiment was repeated three times. Data were analyzed by a one-way ANOVA with Bonferroni’s post test (*** *p* < 0.0001; * *p* < 0.05; ** *p* < 0.001).

In addition to the MTT assay, a clonogenic assay was performed to evaluate the cytotoxic potential of EO MT in CRPC cells. The cells were treated with EO MT (400 µg/mL) for 48 h; then, the essential oil was removed, and the cells were washed and left to grow in the absence of treatment for 12 days. The size and number of colonies were then analyzed. Figure 3a shows that untreated control cells grew forming colonies, whereas EO MT-treated cells developed very few and small colonies, supporting a cytotoxic effect of EO MT. The anticancer activity of several EOs in prostate cancer cell lines involves the induction of apoptosis [4,44].

To investigate the cell death mechanism associated to EO MT cytotoxic activity, DU145 and PC3 cells were treated with EO MT (400 µg/mL) for 48 h and 72 h, and an Annexin V/PI assay was performed. The cytofluorimetric analysis, shown in Figure 3b, indicates that at both times evaluated, the percentage of live cells decreased significantly after EO MT treatment, and the percentages of cells in early and late apoptosis increased. It is interesting to observe that in both cell lines, the percentage of cells in necrosis are unchanged after treatment, demonstrating that, despite the high dose used in this experiment, the EO MT induces a specific apoptotic cell death and not an aspecific necrotic cell death in DU145 and PC3 cells. 

Hence, this result indicates the involvement of an apoptotic process in EO MT-induced CRPC cell death. 

To confirm the apoptosis induction by EO MT in our cells, we performed Western blotting to evaluate the protein expression of cleaved caspase 3, the final effector of the apoptotic pathway, and its molecular target PARP (Poly-ADP-ribose-polymerase), a nuclear enzyme involved in DNA repair processes. The data reported in Figure 3c show an increased expression of cleaved caspase 3 and cleaved PARP in both CRPC cells treated with OE MT, compared to the control cells. EO MT induced a stronger cleavage of the two proteins tested in DU145 cells compared to PC3 cells, confirming what was already observed in the flow cytometric analysis. Finally, to support the proapoptotic role of EO MT in CRPC cells, an MTT assay of treated cells in the presence or absence of pan caspase inhibitor z-VAD was performed. As reported in Figure 3d, EO MT showed a marked cytotoxic effect; z-VAD alone had no effects on cell viability, while when given 4 h before the 48 h EO MT treatment and left for the duration of the treatment, it significantly counteracted the EO MT cytotoxic effect. The literature reported that myrtucommulone, a compound derived from the leaves of *Myrtus communis*, exerts proapoptotic effects by the activation of caspases 9 and 3 and the induction of cytochrome c release from mitochondria into the cytoplasm, causing PARP cleavage and DNA fragmentation [45]. Overall, these data demonstrate for the first time that EO MT exerts a cytotoxic/proapoptotic role in CRPC cells.

#### 2.3.2. Antimigratory Activity of EO MT in CRPC Cells

**Figure 4:**
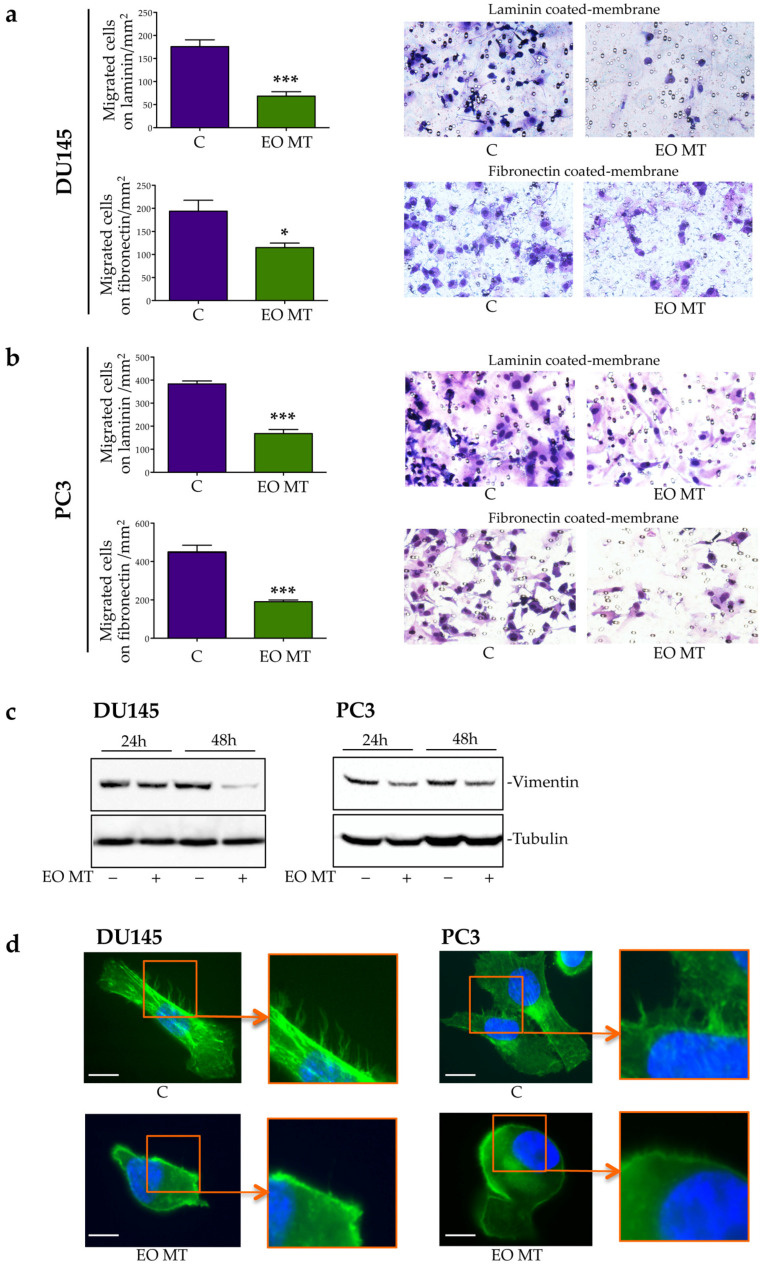
Effect of *Myrtus communis* subsp. *tarentina* (L.) Nyman (EO MT) on cell migration, vimentin expression, and actin rearrangement of DU145 and PC3 cells. Cellular motility of DU145 (**a**) and PC3 (**b**) cells was analyzed by Boyden’s chamber assay. The migratory capacity of control (C) and EO MT (400 µg/mL)-treated cells was analyzed both on laminin-coated and fibronectin-coated membranes. Representative images of migrated cells in each condition are shown ((**a**,**b**) on the right). The graph on the left reports the quantification of migrated cells/mm^2^. Data were analyzed by a one-way ANOVA with Student’s post test (* *p* < 0.05 vs. C and *** *p* < 0.0001 vs. C). Western blot analysis of vimentin (**c**) in CRPC cells treated with EO MT (400 µg/mL) for 24 h and 48 h was performed. Tubulin was used as a loading control.

Cancer metastasis represents one of the most aggressive aspects of tumor development, and cell migration is recognized as a critical step in the metastasis process. To evaluate the effects of EO MT on CRPC migration ability, a Boyden’s chamber migration assay was performed. The migrated cells were quantified after hematoxylin/eosin staining of the membranes. In Figure 4a,b (left side), a quantitative analysis indicated that the DU145 and PC3 cells’ migration was significantly reduced after EO MT treatment both on laminin and fibronectin chemoattractants. Figure 4a,b (right side) shows coated and stained membranes, which highlight that the number of migrated cells after EO MT treatment was lower than in the control group.

Since laminin is a protein present in the epithelial cell matrix, while fibronectin is present in the mesenchymal cell matrix, these data could indicate that DU145 and PC3 cells had the potential to partially modify their epithelial features to develop mesenchymal ones. Indeed, the epithelial-to-mesenchymal transition (EMT) process defines a series of events through which epithelial tumor cells could acquire mesenchymal phenotypes that confer the ability to detach from the primary tumor and to enhance cell motility [46]. EMT is identified by a decrease in epithelial markers, followed by a concurrent increase in mesenchymal markers such as vimentin [47]. In Figure 4c, the Western blot analysis shows a decrease in vimentin protein expression both in DU145 and in PC3 cells after EO MT treatment. Moreover, the migration of cancer cells requires the formation of actin-rich protrusions such as lamellipodia and filopodia, which allow cell motility [48]. Using immunofluorescence, we then analyzed the effect of treatment with EO MT on actin cytoskeleton organization. As reported in Figure 4d, DU145- and PC3-untreated cells expressed filopodia. Treatment with OE MT markedly reduced the expression of these actin protrusions, demonstrating that the antimigratory effect of EO MT, described in previous Boyden’s chamber assay, can also be related to actin cytoskeleton rearrangement. The results presented in Figure 4 indicated, for the first time, that treatment with EO MT reduced CRPC cellular migration, decreasing EMT and modifying actin organization.

In agreement with our observations, Niu and colleagues [49] demonstrated a proapoptotic, antimigratory, and anti-invasive activity of *Croton tiglium* EO, a shrubby plant of the Euphorbiaceae family, in lung cancer cell lines. An antiproliferative and antimigratory effect of EOs extracted from the peel of a specific quality of orange (‘Gannanzao’) was also observed in human cancer cell lines of liver (HepG2) and colon (HCT116) [50]. Similarly, *Erythrina* leaf extract (coral tree) EO exerted antimigratory activity in different human breast cancer cell lines (MDA-MB-231 and MCF-7) [51].

An immunofluorescence analysis was performed to evaluate actin rearrangement (d) in DU145 and PC3 cells treated with EO MT (400 µg/mL) for 24 h. The cells were then fixed and stained with phalloidin (green). Nuclei were stained with DAPI (blue). *Scale bar = 20 μm*. One representative of three different experiments performed is shown. 

Under these experimental conditions, the potential cytotoxic activity of each compound present in EO MT was not evaluated in DU145 and PC3 cell lines; therefore, it is not possible to say which of these compounds are responsible for the observed effects. 

In any case, the observed activity can be assumed to be mediated by the synergy of action of the compounds present in EO MT and described in the literature for their activity similar to the objectives of this work (see Section 2.1).

## 3. Materials and Methods

### 3.1. Plant Material

*Myrtus communis* L. subsp. *tarentina* was cultivated at the Ghirardi Botanic Garden, Department of Pharmaceutical Sciences, University of Milan (Toscolano Maderno, BS). Prof. G. Fico and C. Giuliani identified the plant according to Pignatti et al. [20].

Leaves were concurrently collected for the phytochemical and micromorphological analyses in July 2018 and air-dried at room temperature in the dark for 20 days. Voucher specimens were labeled with the codes GBG2018/060 and GBG2018/061 and deposited in the Herbarium of the Ghirardi Botanic Garden of the University of Milan.

### 3.2. Chemicals and Antibodies

Solvents and reagents were purchased from Sigma Aldrich (Merck group, Milan, Italy) and used as received. The solvents were used with a chromatography grade of purity, while all the reagents had reagent grade purity.

For the Western blot analysis, the following antibodies were utilized: cleaved caspase 3 (#9664), cleaved PARP (#5625), vimentin (#5741), and rabbit Horseradish peroxidase-conjugated secondary antibody (Cell Signaling Technology Inc., Boston, MA, USA). Tubulin (T6199) was from Sigma-Aldrich (St. Louis, MO, USA). Enhanced chemiluminescence reagents were from Cyanagen (Bologna, Italy.) The pan-caspase inhibitor carbobenzoxy-valyl-alanyl-aspartyl-[*O*-methyl]-fluoromethylketone (z-VAD) was from R&D System Inc. (Minneapolis, MN, USA). Dimethyl sulfoxide (DMSO) and 3-(4,5)-dimethylthiazol-2-yl-2,5-diphenyltetrazolium bromide (MTT) were purchased from Sigma-Aldrich (St. Louis, MO, USA). The annexin V-FITC/PI apoptosis detection kit was from eBioscience (1030 Vienna, Austria).

### 3.3. Cell Cultures

The human CRPC cell lines DU145 and PC3 were from American Type Culture Collection (ATCC, Manassas, VA, USA). The PNT1A cell line was established by the immortalization and cloning of normal prostate epithelial cells with SV40 large T antigen [51]. Cells were cultured in RPMI medium supplemented with FBS (5% for DU145) (7.5% for PC3) (10% for PNT1A), glutamine (1 mmol/L) and antibiotics (100 IU/mL of penicillin G sodium and 100 μg/mL of streptomycin sulfate). Cells were cultured in humidified atmosphere of 5% CO_2_/95% air at 37 °C.

### 3.4. Preparation of Essential Oil

Leaves were weighted, grounded, placed into a 4 L flask filled with deionized water in order to cover all the material inside, and then subjected to hydrodistillation using a Clevenger-type apparatus for 2 h. Once obtained, the pale-yellow essential oil was decanted and separated from water, with the residual drops removed using anhydrous sodium sulphate. The oil yield was estimated on a dry weight basis (*w*/*w*).

The results were expressed as the mean of the values obtained for 3 replications.

The essential oil obtained was used both for phytochemical analyses and for the determination of biological activities.

### 3.5. GC-MS Analysis of Essential Oil

The essential oil was analyzed by GC-MS using a Thermo Scientific TRACE ISQ QD Single Quadrupole instrument. EO separation was performed by a capillary column VF-5ms (5% phenyl-methyl-polisiloxane, length 30 m, 0.25 mm i.d., 0.1 μm film thickness); the temperature gradient was: 8 min at 50 °C, then 4 °C/min till 60 °C, then 6 °C/min from 60 °C to 160 °C, and finally, 20 °C/min from 160 °C to 280 °C. Injector and detector temperatures were set to 280 °C; carrier gas He, flux 1 mL/min: the mass range detected was 50–500 *m*/*z*. EO was analyzed diluted 1:100 with n-hexane, with an injection volume of 1 µL.

Mass spectra were analyzed by the Wiley Mass Spectra Library, NIST Mass Spectral Search Program, and NIST Tandem Mass Spectral library 2.3; compounds were identified by mass fragmentation and retention index, compared with data stored in mass databases (WILEY, NIST18). The complete chromatogram of EO and MS spectra of compounds **3** and **27** (Table 1) were reported as Supplementary Materials.

### 3.6. Light Microscopy and Fluorescence Microscopy

The micromorphological investigation under light microscopy and fluorescence microcopy was performed on leaves collected from the same individual. We observed both fresh material and fixed samples included in historesin (Technovit^®^ 7100, Kulzer Technik, Wehrheim, Germany). 

For the fresh material, sections of thickness ranging from 30 to 50 µm were obtained using a vibratome.

The samples were also fixed in F.A.A. solution (Formaldehyde:Acetic Acid:Ethanol 70% = 5:5:90) for 48 h at 4 °C. Subsequently, the fixed samples were washed in 70% ethanol for 24h; they were then dehydrated progressively by treatment with 80% ethanol for 2 h, 95% ethanol for 2 h, and then twice in absolute ethanol for 2 h/each. Pre-inclusion was then performed first with ethanol and historesin in a 1:1 ratio for one night, then with a 1:2 ratio for 2 h and in pure historesin for 3 h. Finally, the inclusion was carried out in a polypropylene capsule with the addition of hardener in a ratio of 1:15 of basic resin. The historesin samples were cut in 2 µm sections by an ultramicrotome.

At least 10 replicates were gathered to assess the variability level in the morphology, distribution, and histochemistry of the secretory cavities. The following dyes were employed [19]: Fluoral Yellow-88 for total lipids; Nile Red for neutral lipids; Nadi reagent for terpenes; Alcian Blue for mucopolysaccharides; Ferric Trichloride for polyphenols; Aluminum Trichloride and Naturstoff reagent A for flavonoids.

### 3.7. MTT Viability Assay

All cells were seeded in 24-well plates—DU145 and PC3 cells at a density of 3 × 10^4^ cells/well, whereas PNT1A cells at a density of 4 × 10^4^ cells/well. After 24 h, the cells were exposed to EO MT at different concentrations (25, 50, 100, 200, 400 μg/mL) and incubated for 24 h, 48 h, and 72 h (DU145 and PC3 cells). The PNT1A cells were incubated with OE MT for 48 h. The DU145 and PC3 cells were exposed to EO MT (400 μg/mL) for 48 h in the presence or absence of the pan caspase inhibitor z-VAD (50 μM). After the treatments, the medium was changed with MTT solution (0.5 mg/mL) in RPMI without phenol red and FBS. After 30 min of incubation, the appeared formazan precipitate was dissolved with isopropanol. Absorbance at 550 nm was measured through an EnSpire Multimode Plate reader (PerkinElmer, Milan, Italy).

### 3.8. Clonogenic Assay

The DU145 and PC3 cells were seeded at a density of 2 × 10^2^ or 1.5 × 10^2^ cells/2ml in 6-well plates. After 24 h, the cells were exposed to EO MT at concentration of 400 μg/mL for 48 h. The cells were then cultured for 12 days, changing the medium twice a week. Samples were then fixed using a methanol solution and stained with crystal violet 0.15%. A Nikon digital camera captured colony formation images, and the colonies were counted.

### 3.9. Annexin V/Propidium Iodide Assay

The cell death analysis of DU145 and PC3 cells treated with EO MT was determined using the Annexin V/propidium iodide (PI) apoptosis kit. The assay was performed according to Cristofani and coworkers (Cristofani et al., 2018). The DU145 and PC3 cells were seeded at a density of 1.5 × 10^5^ cells/dish in 6 cm Petri dishes for 24 h, and then exposed to EO MT (400 μg/mL) for 48 h or 72 h. The cells were then collected and resuspended in 200 μL of binding buffer followed by staining with Annexin-V and PI, as recommended in the manufacturer’s protocol. A flow cytometric analysis was performed with Novocite 3000 (Acea Bioscience, Inc) and the data analyzed by software Novo Express.

### 3.10. Western Blot Assay

For the Western blot analysis, the DU145 and PC3 cells were seeded in 60 mm dishes at a density of 2 × 10^5^ cell/dish and treated with EO MT (400 μg/mL) for 24 h or 48 h. At the end of the treatment, the cells were lysed with RIPA buffer (0.05 mol/L of Tris HCl pH 7.7, 0.15 mol/L of NaCl, 0.8% SDS, 10 mmol/L of EDTA, 100 μM/L of NaVO_4_, 50 mmol/L of NaF, 0.3 mmol/L of PMSF, 5 mmol/L of iodoacetic acid) containing leupeptin (50 μg/mL), aprotinin (5μL/mL), and pepstatin (50 μg/mL). An amount of 30 μg of protein extract was separated through SDS gel electrophoresis and transferred to 0.45 μm nitrocellulose membranes (Amersham, Cytiva). After blocking with non-fat dried milk, the membranes were incubated at 4 °C overnight using the specific antibodies: rabbit anti cleaved caspase 3 (1:500), rabbit anti cleaved PARP (1:1000), rabbit anti-vimentin (1:1000), mouse anti-alpha-tubulin (1:2000). Peroxidase-conjugated secondary anti-rabbit or anti-mouse antibodies were used for 1 h at room temperature; the membranes were then processed using the enhanced chemiluminescence kit Cyanagen Ultra (Cyanagen, Srl, Bologna, Italy). Tubulin expression was utilized as a loading control.

### 3.11. Migration Assay

The migratory capacity of the DU145 and PC3 cells treated with EO MT was evaluated by using a 48 well-Boyden chamber (NeuroProbe, Inc.; Gaithersburg, MD, USA) containing a polycarbonate membrane with a pore diameter of 8 μm (Nucleopore, Concorezzo, Milan, Italy). The membranes were coated with 50 μg/mL of laminin (Sigma, L2020) or 50 μg/mL of fibronectin (F0895) on their lower side. A volume of 50 μL of control or treated cell suspensions (in medium without FBS) was placed in each well on the upper part of the chamber at a concentration of 1.5 × 10^5^ cells/50 μL. The cells were incubated at 37 °C in a humidified atmosphere of 5% CO_2_/95% air and allowed to migrate through coated membranes for 3 h. After this time, the lower side of the membrane was fixed and stained, and the number of migrated cells was determined by count using standard optical microscopy (20×). The results of three different experiments of migration are presented. Each experimental group consisted of 12 samples.

### 3.12. Immunofluorescence

Immunofluorescence with phalloidin was used to examine the actin cytoskeleton of the DU145 and PC3 cells after EO MT treatment. For immunofluorescence, the cells were seeded on polylisine-coated 13 mm glass coverslips at a density of 3 × 10^4^ cell/well in 24-well plates for 24 h before treatments. After the treatment with a 400 μg/mL concentration of EO MT for 24 h, the cells were fixed with 4% formaldehyde and 3% sucrose for 10 min at room temperature and washed in PBS for 3 × 5 min. Then, Phalloidin-iFluor 488 was added to each well and incubated for 15 min at 37 °C in a humidified atmosphere of 5% CO_2_/95% air. After being washed in PBS for 3 × 5 min, the cell nuclei were stained with DAPI. Coverslips were then mounted on glass slides using mounting medium (Mowiol) and, finally, fluorescence images were captured with a Zeiss Axiovert 200 microscope with a 63×/1.4 objective lens linked to a Coolsnap Es CCD camera (Roper Scientific-Crisel Instruments, Rome, Italy). 

### 3.13. Statistical Analysis

The statistical analysis was performed with a statistic package (GraphPad Prism 5, GraphPad Software San Diego, CA, USA). Values are represented as the mean ± SEM. Differences between groups were analyzed using a one-way ANOVA followed by Student’s, Dunnett’s, or Bonferroni’s post hoc tests. Each experiment was performed three times.

## 4. Conclusions

We evaluated for the first time the effect of EO MT on two CRPC cell lines. Our data suggest that EO MT exerts cytotoxic and antimigratory activity in CRPC cells and is a possible promising source of anticancer compounds. For this reason, these results open the possibility of further studies aimed at analyzing which compounds are responsible for the activity observed for EO MT.

The compounds of the terpene profile of *M. communis* subsp. *tarentina*, with high relative abundance and/or already described in the literature for their biological activity similar to the goals of this work, arouse our attention. Future studies will allow us to understand whether the activity detected should be ascribed to the synergy of action of the compounds occurring in the terpenic profile, or whether it is specifically mediated by some of them.

## Figures and Tables

**Table 1:** GC-MS profile of the leaf essential oil of *Myrtus communis* subsp. *tarentina*.

*N.*	Class	Component	LRI ^a^	RI ^b^	%	SD
*1*	MH	α-pinene	914	937	17.73	0.19
*2*	MH	β-pinene	975	979	0.38	0.09
*3*	MO	Unidentified	1029	-	11.96	0.49
*4*	MH	γ-terpinene	1050	1060	1.05	0.25
*5*	MH	Terpinolene	1077	1088	1.32	0.39
*6*	MO	Linalool	1093	1099	6.16	0.20
*7*	MO	Pinocarveol	1130	1139	0.42	0.03
*8*	MO	α-pinocarvone	1147	1164	0.16	0.02
*9*	MO	4-terpineol	1160	1177	1.28	0.13
*10*	MO	α-terpineol	1172	1189	7.36	0.38
*11*	MO	linalyl acetate	1248	1257	1.54	0.06
*12*	MO	*trans*-geraniol	1256	1255	1.09	0.20
*13*	MO	*trans*-pinocarvyl acetate	1291	1297	0.15	0.02
*14*	MO	methyl geranate	1312	1324	1.76	0.12
*15*	MO	exo-2-hydroxycineole acetate	1332	1344	0.22	0.00
*16*	MO	α-terpinyl acetate	1340	1350	1.02	0.05
*17*	MO	C-acetylsyncarpic acid	1350	1369	1.53	0.06
*18*	MO	geranyl acetate	1370	1382	5.92	0.06
*19*	MO	methyl eugenol	1395	1402	4.34	0.62
*20*	SH	β-caryophyllene	1410	1419	5.17	0.20
*21*	NH	5-hydroxy-2,2,6,6-tetramethyl-4-propionylcyclohex-4-ene-1,3-dione	1444	1464	0.50	0.09
*22*	SH	α-humulene	1451	1454	12.39	1.14
*23*	NH	trans-β-ionone	1473	1486	0.34	0.01
*24*	SH	α-farnesene	1493	1508	0.28	0.01
*25*	NH	Durohydroquinon ^c^	1507	-	7.26	0.65
*26*	SO	caryophyllene oxide	1576	1581	1.59	0.07
*27*	SO	Unidentified	1591	-	0.78	0.04
*28*	SO	humulene epoxide II	1602	1606	3.19	0.24
*29*		Unidentified	1610		1.46	0.13
		**Monoterpene hydrocarbons**			**20.49**	**0.54**
		**Oxygenated monoterpenes**			**32.95**	**1.63**
		**Sesquiterpene hydrocarbons**			**17.84**	**1.32**
		**Oxygenated sesquiterpenes**			**4.78**	**0.31**
		**Non-terpene hydrocarbons**			**8.10**	**0.75**
		**Non identified compounds**			**14.19**	**0.34**
		**Total compounds**			**98.36**	**0.08**

LRI ^a^ = Linear Retention Index, experimentally obtained on a VF-5ms column using a C_7_–C_30_ mixture of *n*-alkanes. RI ^b^ = Retention Indexes as reported in NIST and Wiley databases, used as reference. ^c^ = tentatively identified.

## Data Availability

Not applicable.

## Supplementary Materials

The following supporting information can be downloaded at: https://www.mdpi.com/article/10.3390/plants12061293/s1, The EO GC-MS chromatogram and MS spectra for compounds **3** and **27** (Table 1) are available as Supplementary data.
