# Supplementary material for: The Leaf Essential Oil of Myrtus communis subsp. tarentina (L.) Nyman: From Phytochemical Characterization to Cytotoxic and Antimigratory Activity in Human Prostate Cancer Cells
Source: Plants (Basel). 2023 Mar 13;12(6):1293. doi: 10.3390/plants12061293 (PMC10056649; doi:10.3390/plants12061293)

Chromatogram of essential oil obtained from *Myrtus tarentina*

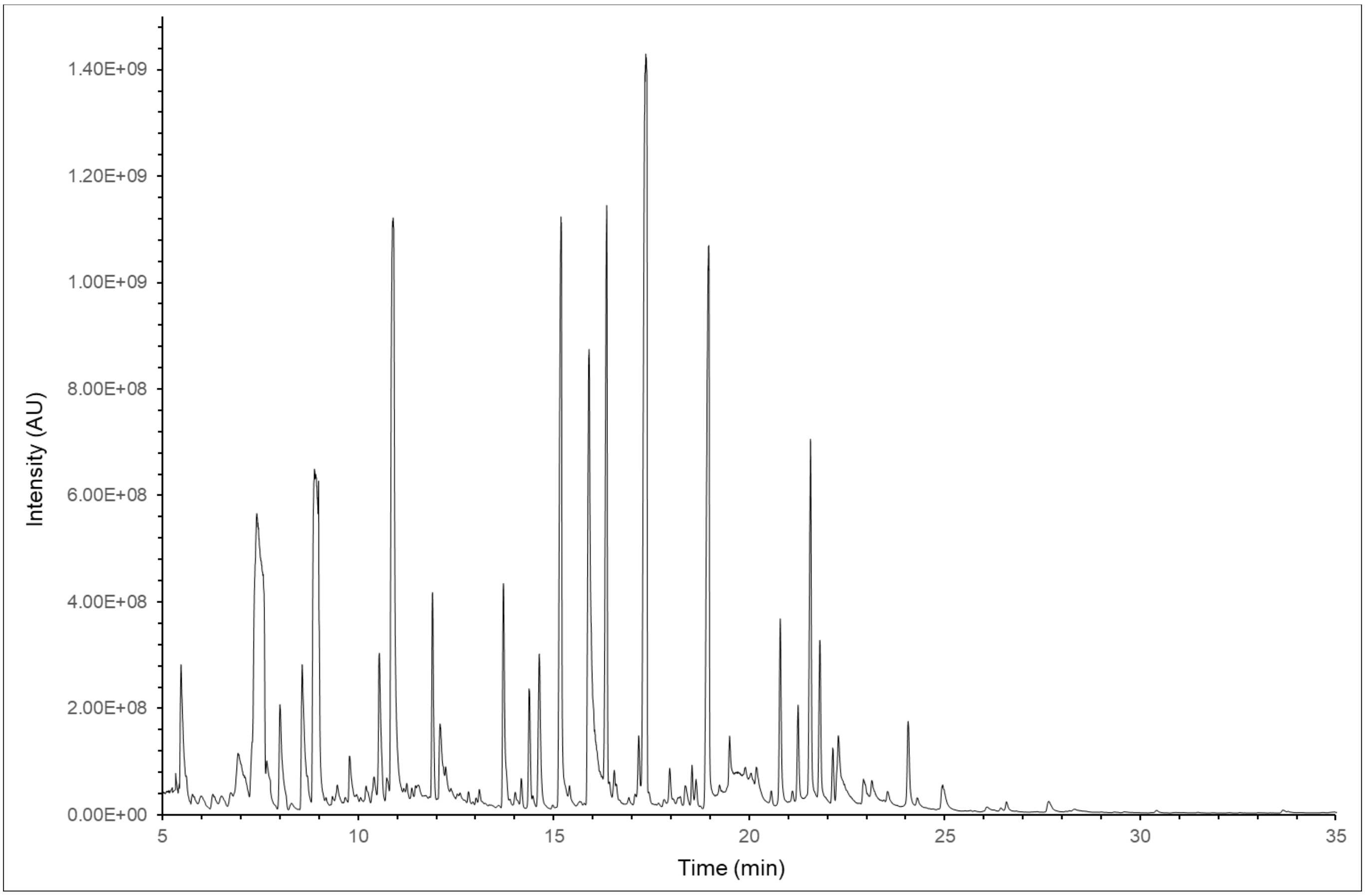

MS spectrum of peak with RT =7.42 min

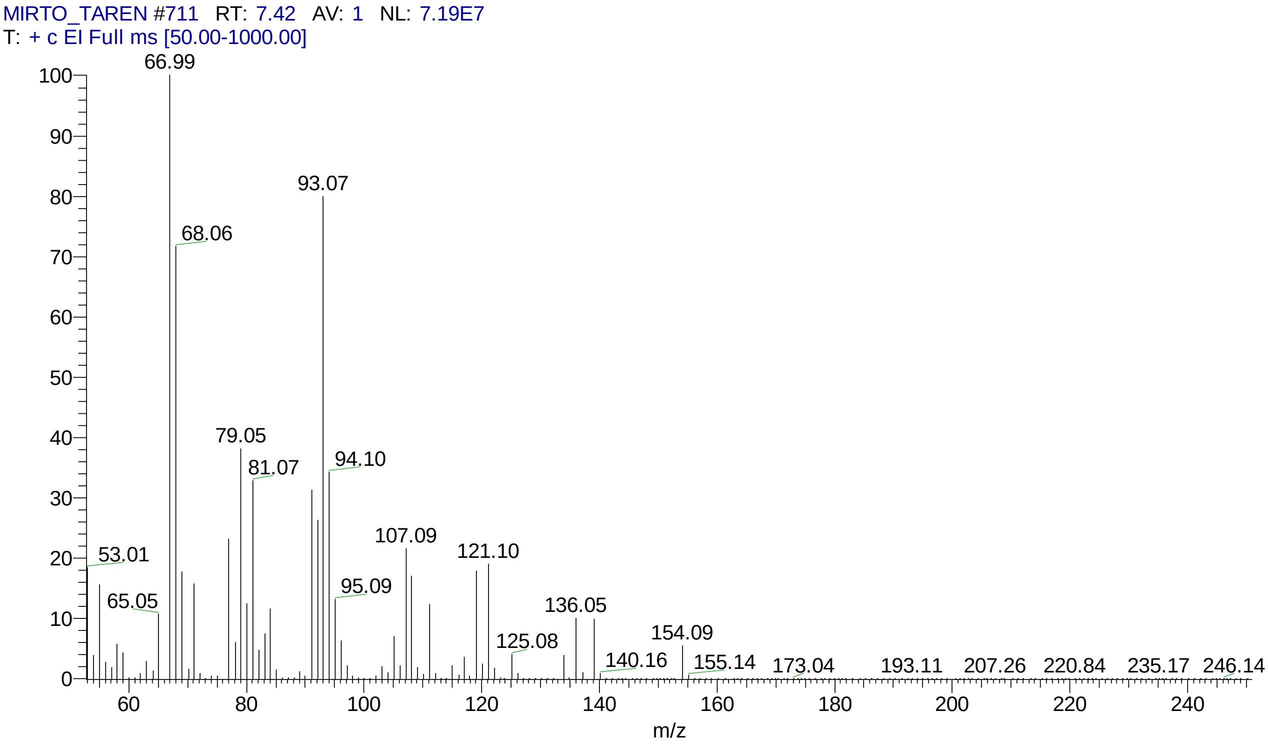

MS Spectrum of peak with RT = 21.24 min

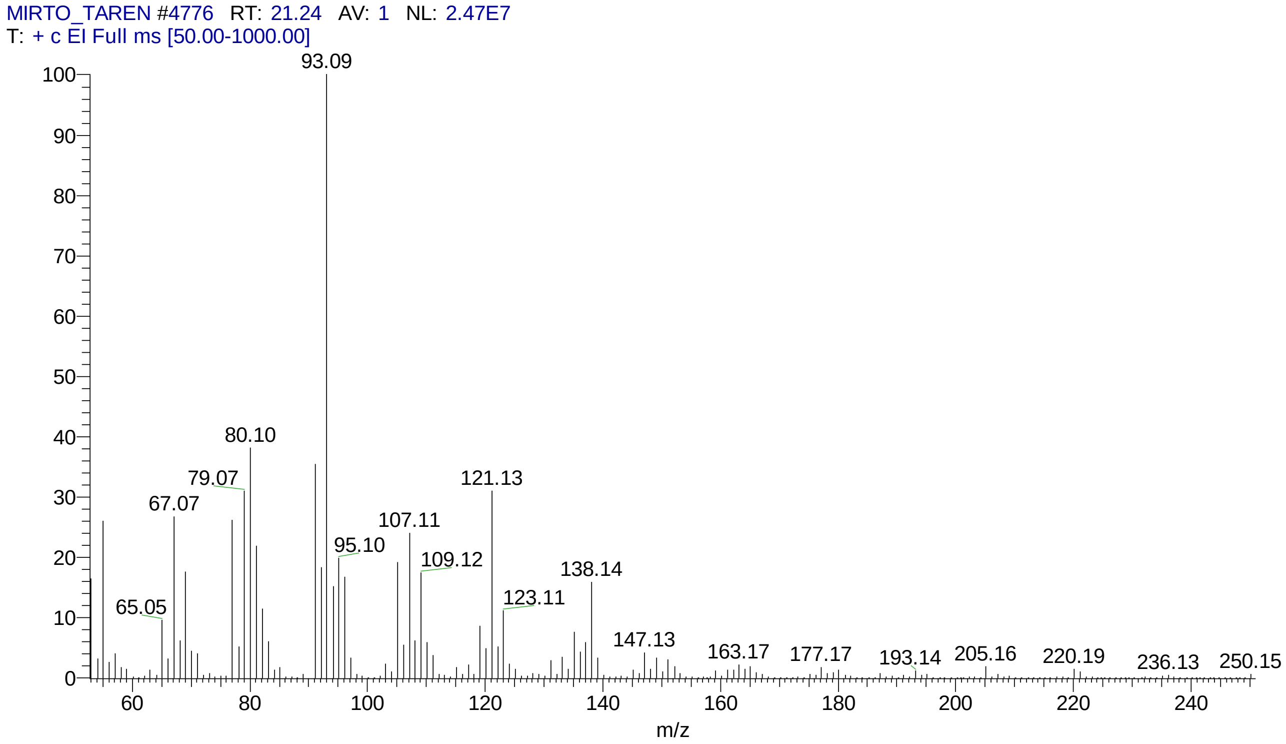

Supplement: Supplementary file 1 [file plants-12-01293-s001.zip › plants-2254642-supplementary.pdf]
